# Syringocystadenoma papilliferum in a 20-year-old adult: a case report and literature review

**DOI:** 10.1093/jscr/rjac470

**Published:** 2022-10-25

**Authors:** Valeska Siulinda Candrawinata, Heru Sutanto Koerniawan, Patricia Diana Prasetiyo, Bernard Agung Baskoro

**Affiliations:** Faculty of Medicine, Pelita Harapan University, Tangerang, Indonesia; Department of Surgery, Faculty of Medicine, Pelita Harapan University, Siloam General Hospital, Tangerang, Indonesia; Department of Pathology Anatomy, Faculty of Medicine, Pelita Harapan University, Siloam General Hospital, Tangerang, Indonesia; Division of Oncology, Department of Surgery, Faculty of Medicine, Pelita Harapan University, Siloam General Hospital, Tangerang, Indonesia

## Abstract

Syringocystadenoma papilliferum is a rare, benign hamartomatous neoplasm of skin adnexal originating from pluripotent cells differentiating into either apocrine or eccrine sweat glands. It usually appears at birth, during infancy or puberty and commonly located at head and neck. This case report illustrates a rare occurrence at an atypical anatomical location and unusual onset. In this case report, we report a 20-year-old female with a chief complain of solitary pink-brown color fleshy plaque with soft-medium consistency on her left flank region since the last 7 months. She underwent complete surgical excision and histopathology examination, which confirmed the diagnosis as syringocystadenoma papilliferum without sign of malignancy, with main characteristics histologically include cystic invaginations from the epidermis lined by double layers of epithelial and myoepithelial cells. Despite having benign characteristics, rare transformations to malignancy have been reported. Therefore, complete surgical excision and histopathology examination should be done in suspicion of syringocystadenoma papilliferum.

## INTRODUCTION

Syringocystadenoma papilliferum is a rare, benign hamartomatous neoplasm of skin adnexal originating from pluripotent cells differentiating into either apocrine or eccrine sweat glands. Approximately half of its occurrence is found at birth. Its clinical characteristics are non-specific and so histopathology remains a gold standard for diagnosis. Due to risk of a malignant change, complete surgical excision is the treatment of choice. Here, we present a case of syringocystademona papiliferum at an atypical anatomical location and rare onset.

## CASE PRESENTATION

A 20-year-old female visited the surgical outpatient department with chief complaint of a mass on her left flank region since the last 7 months that was slowly increasing in size and sometimes oozed fluid. The patient also complains of occasional pruritus and mild tenderness. The patient did not notice any other lesion. Patient reported no significant personal and family history of any disease, medication and/or genetic condition.

**Figure 1:**
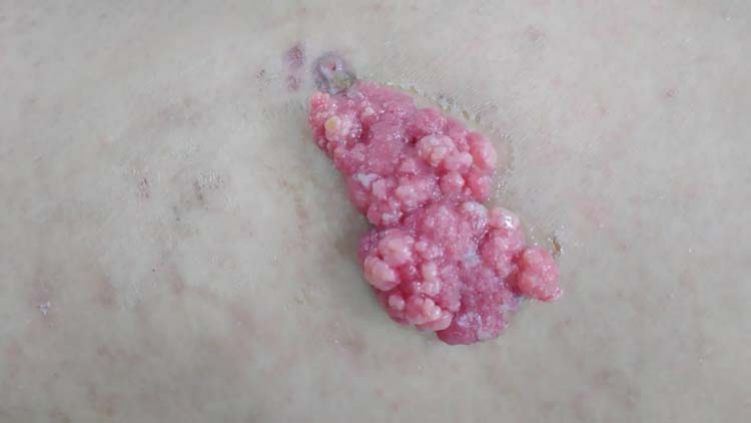
A solitary mass, with pink-brown color, measurement of 6.5 × 3 × 2 cm, soft-medium consistency at left flank region.

Physical examination—including vital signs—was within normal limits. Cutaneous examination of the left flank region revealed a solitary pink-brown color fleshy plaque with central crustation, measurement of 6.5 × 3 × 2 cm, with a soft-medium consistency (Fig. 1).

Pre-operative laboratory and imaging were unremarkable. The patient was administered prophylaxis antibiotic: 1 g of Ceftriaxone via intravenous 1 h before incision.

The patient underwent surgery by an experienced general surgeon at a private general hospital. The surgery was done with the patient in prone position, under general anesthesia. The lesion was excised completely with 1-cm margin and sent to histopathological examination. Gross specimen demonstrated a solitary mass with pink-brown color, measurement of 6.5 × 3 × 2 cm, soft-medium consistency (Fig. 1).

**Figure 2:**
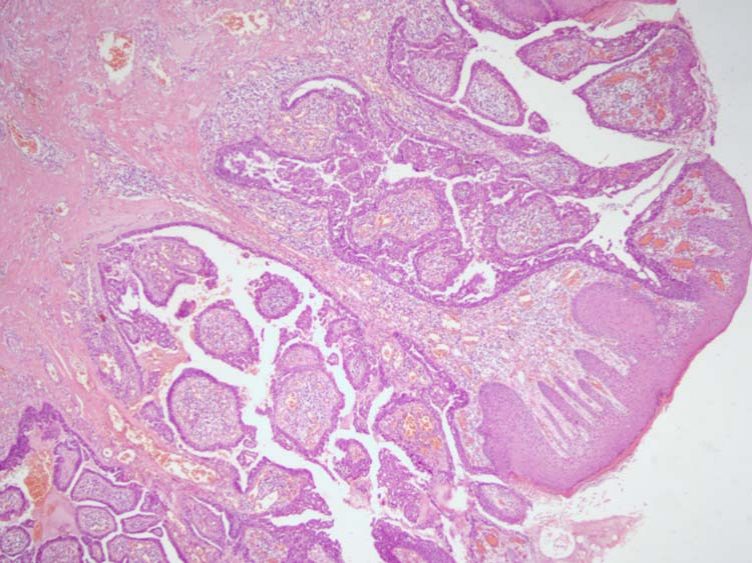
H&E staining, 4× magnification. Cystic invaginations of the infundibular epithelium projecting into the dermis, covered by a double cell layer.

**Figure 3:**
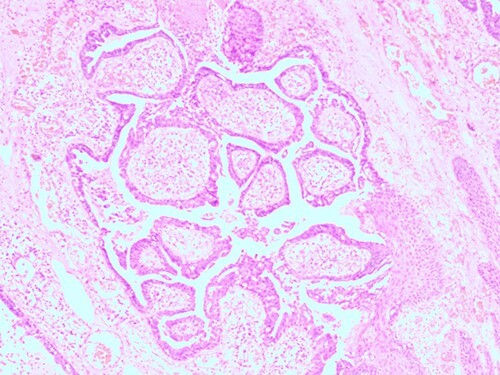
H&E staining, 4× magnification. Verrucous (papillomatous) epidermal hyperplasia with hyperkeratosis and hypergranulosis.

**Figure 4:**
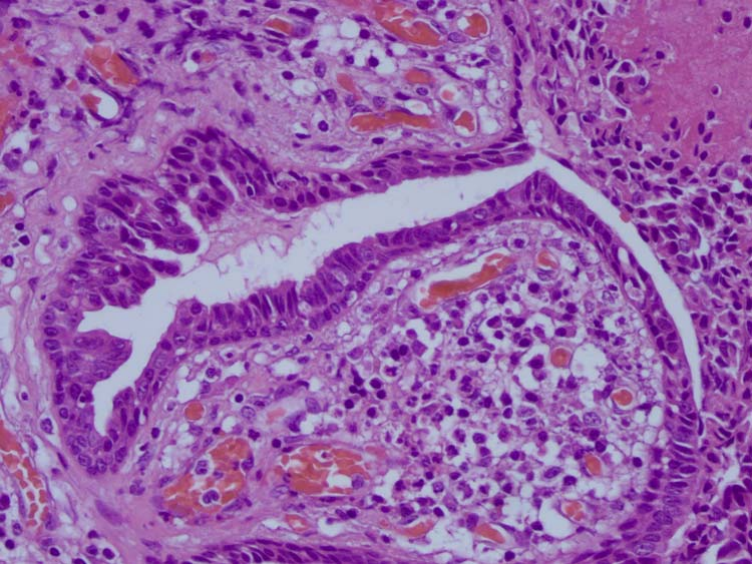
H&E staining 10× magnification. Proliferation of glands with prominent papillary architecture.

**Figure 5:**
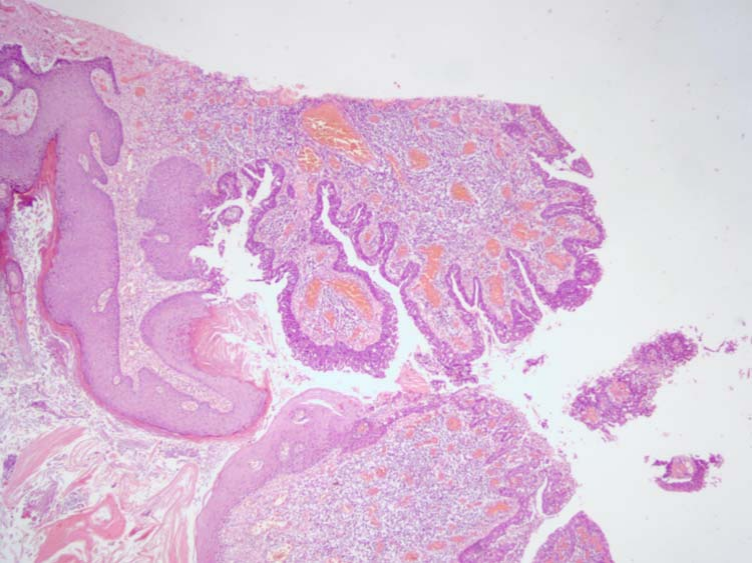
H&E staining 10× magnification. Irregular duct-like structures and cystic spaces.

**Figure 6:**
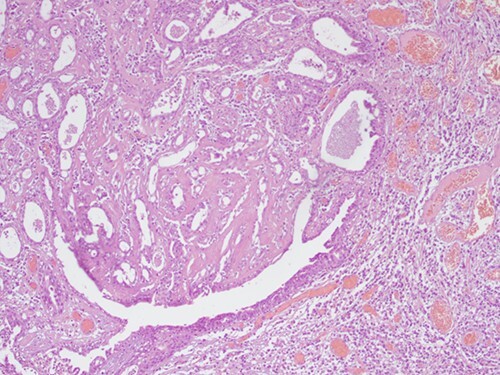
H&E staining 40× magnification. Fibrous cores of papillae contain numerous plasma cells and are lined by a double layer of cuboidal columnar epithelium.

**Figure 7:**
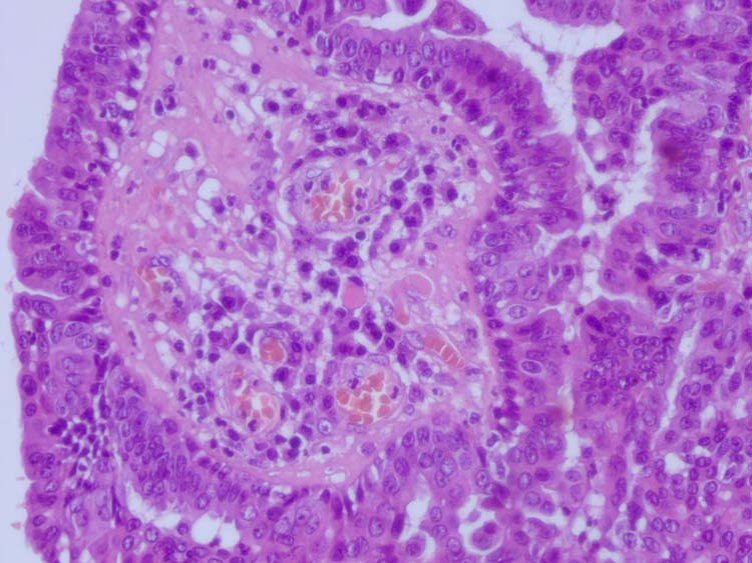
H&E staining 40× magnification Glands with double layer of cuboidal columnar epithelium and numerous stromal plasma cells.

The histopathological examination found cystic invaginations of the infundibular epithelium projecting into the dermis, covered by a double cell layer (Fig. 2), proliferation of glands with prominent papillary architecture (Fig. 3) and fibrous cores containing numerous stromal plasma cells (Fig. 4). Other findings include verrucous (papillomatous) epidermal hyperplasia with hyperkeratosis and hypergranulosis (Fig. 5), irregular duct-like structures and cystic spaces (Fig. 6) and glands with double layer of cuboidal columnar epithelium and numerous stromal plasma cells (Fig. 7). These findings are compatible with Syringocystadenoma papilliferum with no signs of malignancy.

Based on clinical symptoms and histopathological examination, diagnosis of syringocystadenoma papilliferum was confirmed.

After surgery, the patient stayed in hospital for 1 day and complained of mild pain at excision area. The patient was discharged with medications: Co-Amoxiclav 625-mg TID and Paracetamol 500-mg TID. Follow-up was done 3 and 7 days after surgery at outpatient department with adequate wound healing without any complications.

## DISCUSSION

Syringocystadenoma papilliferum is a rare, benign hamartomatous skin adnexal neoplasm that arises from pluripotent cells. This rare neoplasm is derived from apocrine or eccrine sweat glands. Most of its occurrence appears at birth or infancy and ~15–30% appear around puberty [1]. Study from Lee *et al*. showed that syringocystadenoma papilliferum are most often located at head and neck area [2, 3]. Other sites include arms, face, chest, abdomen, arms, thighs and perineum [4]. This case had a rare onset of appearance, which was at adulthood and also a very rare location: at left flank region.

Its clinical characteristics are non-specific with high variability of appearance and size ranging from a few millimeters to several centimeters [1,5]. The slowly growing fleshy plaque may sometimes ooze fluid or bleed [6]. There are three clinical types described: Plaque (located at scalp as an alopecic patch and may grow in size during puberty becoming nodular, verrucous or crusted), Solitary nodule (domed nodules 5–10 mm in size with peduncle, located at trunk, shoulder and axilla) and extremely rare type: Linear (multiple firm papule or umbilicated nodule 1–10 mm in size, pink-red in color, located at face and neck) [1, 4].

Different types of adnexal origin tumor have been reported to appear in concurrence. Chauhan *et al*. reported three cases of linear syringocystadenoma papilliferum associated with nevus sebaceous, tubular apocrine adenoma, apocrine cystadenoma and hidrocystoma [4]. Lee *et al*. showed that some syringocystadenoma papilliferum are not associated with a previously benign tumor [2]. In this case, the tumor was not associated with other tumors.

Histopathology remains the gold standard for the diagnosis of adnexal tumors [4]. Histologically, syringocystadenoma papilliferum is mainly characterized by cystic invaginations from the epidermis lined by double layers of epithelial and myoepithelial cells. Other characteristics include papillomatosis, numerous plasma cells within stroma, irregular papillary architecture and cystic spaces or glands and loss of double-layered epithelium [1, 7].

Syringocystadenocarcinoma papilliferum—the malignant counterpart of syringocystadenoma papilliferum—is asymmetric, poorly circumscribed and often extending deep into the subcutaneous fat [8]. Histopathology shows many resemblances with the main differences being higher ratio of nuclear cytoplasmic, irregularity of nuclear, coarse chromatin and increased mitotic activity in syringocystadenocarcinoma papilliferum [6].

In most cases, adenomatous component have shown positivity for CK7 and CK19 on immunohistochemistry examination and their heterogenous expressions were observed in the luminal cells of eccrine and apocrine dermal ducts [5,9]. Limitation to this case report is unavailability of immunohistochemistry examination.

Although benign, a rare transformation of syringocystadenoma papilliferum to basal cell, metastatic and ductal carcinoma has been reported [10]. Anggrawal *et al*. reported that among 30 cases, 14 cases (46.7%) had an associated contiguous squamous proliferation with the most common clinical presentation was skin-colored to erythematous warty papules [11]. Basal cell carcinoma has been reported to be seen in 10% of patients with syringocystadenoma papilliferum [12].

The treatment of choice for syringocystadenoma papilliferum is complete surgical excision and histopathology to confirm the diagnosis as a benign tumor with a generally good prognosis [1, 13].

## CONCLUSION

Syringocystadenoma papilliferum is a rare, benign hamartomatous neoplasm of skin adnexal that usually appears at birth, during infancy or puberty and commonly located in head and neck area with a non-specific clinical manifestation. This case reported here had a rare onset, at an atypical anatomical location, which serve as new insights for syringocystadenoma papilliferum characteristics. The gold standard for diagnosis is histopathology examination. Despite its benign characteristics, there have been reports of rare transformations to malignancy. Hence, a suspicion of syringocystadenoma papilliferum should be treated with a complete surgical excision.

## PATIENT PERSPECTIVE

At 1-day post-op follow up, the patient felt relieved and content that the tumor had been excised. Later on at 3- and 7-day post-op follow up, the patient was also satisfied as the pain felt and the scar resulted were both minimal.
